# Comprehensive analysis of differentially expressed microRNAs and mRNAs in MDBK cells expressing bovine papillomavirus *E5* oncogene

**DOI:** 10.7717/peerj.8098

**Published:** 2019-11-22

**Authors:** Feng Pang, Zhen Chen, Chengqiang Wang, Mengmeng Zhang, Zhenxing Zhang, Xiaohong Yang, Yiying Zheng, Ang Liu, Yiwen Cheng, Jie Chen, Bin Li, Li Du, Fengyang Wang

**Affiliations:** College of Animal Science and Technology, Hainan University, Hainan Key Lab of Tropical Animal Reproduction & Breeding and Epidemic Disease Research, Hainan University, Haikou, China

**Keywords:** Bovine papillomavirus, E5, microRNAs, RNA-seq, Target genes, miRNA-gene network

## Abstract

Delta bovine papillomaviruses (δBPVs) causes fibropapillomas or bladder cancer in cattle. *E5* is the major oncogene of δBPVs; however, the influence that *E5* oncogene has on host microRNA (miRNA) and mRNA expression profiles remains little elucidated. In the present study, small RNA sequencing and RNA sequencing were used to explore alterations in miRNAs and mRNAs in *E5* over-expressing Madin-Darby bovine kidney (MDBK) cells compared with controls. In total, 77 miRNAs (including 30 bovine-derived miRNAs) and 223 genes were differentially expressed (DE) following *E5* overexpression. The dysregulated genes were mainly involved in metabolic and biosynthetic processes. We constructed a potential miRNA-gene regulatory network from the differentially expressed genes (DEGs) and DE miRNAs. Finally, 22 DEGs and nine DE miRNAs were selected for RT-qPCR validation. Of these, downregulation of six miRNAs, *bta-miR-34c*, *bta-miR-122*, *bta-miR-195*, *bta-miR-449b*, *bta-miR-2425-5p*, and *bta-miR-2428-3p* were confirmed; In addition, upregulation of 16 genes, *ACSS2*, *DDIT4*, *INHBE*, *INSIG1*, *PNRC1*, *PSAT1*, *PSPH*, *PYCR1*, *SC4MOL*, *SLC34A2*, *SCD*, *SPARC*, *IDI1*, *PCK2*, *HMGCS1*, and *SMIM14* and downregulation of two genes, *BATF3* and *WFDC2* were confirmed. Specially, *bta-miR-34c* and *bta-miR-449b* potentially regulated *PYCR1* and *DDIT4*, which were involved in cancer progression and angiogenesis. Our study presented for the first time the comprehensive miRNA and mRNA alterations in MDBK cells expressing the BPV *E5* oncogene, providing new insights into the tumorigenesis induced by BPV E5.

## Introduction

Bovine papillomavirus (BPV) is an oncogenic double-stranded DNA virus that induces benign hyperproliferative lesions in cutaneous and mucous epithelia (Suprynowicz et al., 2005; Tsirimonaki et al., 2006). BPVs are strictly species-specific and only infect their natural hosts. Cross-species infection only occurs in horses and other equids by BPV-1 and BPV-2 (Nasir & Campo, 2008). A total of 14 types of BPV have been researched and classified into four genera based on their biological characteristics and genetic homology (Nasir & Campo, 2008; Roperto et al., 2016). Among these, BPV-1, -2, and -13 (Nasir & Campo, 2008) belong to delta papillomaviruses (δPVs), which are also commonly termed fibropapillomaviruses. Moreover, only δPVs have the ability to infect both the epithelium and the underlying derma, causing fibropapillomas (Munday et al., 2015; Roperto et al., 2016). The BPV genome consists of double-stranded circular DNA of approximately 8,000 base pairs (bp). The genome is divided into three regions: early genes (*E5, E6, E7*, encoding non-structural proteins), late genes (*L1* and *L2*, encoding viral capsid protein) and the long control region (responsible for viral replication and transcription regulation) (Munday, 2014; Nasir & Campo, 2008).

*E5* is the major oncogene of BPV, while *E6* and *E7* are the main oncogene of human papillomavirus (HPV) (Marchetti et al., 2002). BPV type 1 E5, which encodes a 44-amino-acid protein, composed of an amino-terminal 30-amino-acid strongly hydrophobic domain and a carboxyl-terminal 14-amino-acid hydrophilic domain (Cohen et al., 1993; Horwitz et al., 1988). *E5* is mainly localized to membranes of the Golgi apparatus (GA) and endoplasmic reticulum of the host cell (Suprynowicz et al., 2005; Tsirimonaki et al., 2006). Because of its hydrophobic composition, an antiserum generated against the carboxyl-terminal third of the BPV1 E5 protein has been widely used to immunoprecipitate the E5 protein from BPV1-transformed mouse and hamster cells (Horwitz et al., 1988; Nilson & DiMaio, 1993; Schlegel et al., 1986). Previous studies have reported that BPV *E5* transformed cells in vitro by mainly binding to and activating the platelet-derived growth factor (PDGF-) β receptor (DiMaio & Mattoon, 2001; Nilson & DiMaio, 1993). Additionally, BPV *E5* is capable of retaining major histocompatibility class I complex (MHC I) in the GA and prevents its transport to the cell surface (Marchetti et al., 2002). The lack of MHC I on the cell surface prevents the presentation of virus peptides to the cellular immune system. BPV *E5* also binds to the 16 kDa transmembrane subunit of the vacuolar H+-ATPase (V-AT Pase). Thus, V-ATPase activity is inhibited and the pH balance of the GA is disturbed, which influences the activity of some important growth proteins, including PDGFβ and finally gives rise to cellular transformation (DiMaio & Mattoon, 2001).

MicroRNAs (miRNAs) are a large class of endogenous, non-coding, small RNAs that are approximately 22 nucleotides in length and are found in animals, plants, and even in some viruses (Krol, Loedige & Filipowicz, 2010). They play important roles in the post-transcriptional regulation of gene expression by targeting mRNAs at their 3′ untranslated regions (UTRs), giving rise to the cleavage or translation inhibition of their targets (Zhang et al., 2007). They participate in multiple biological processes including cell proliferation, cell apoptosis, and cancer (Cheng et al., 2005; Kumar et al., 2018; Martinez et al., 2008; Murakami et al., 2006). Moreover, they play critical roles in regulating host gene expression during viral infection (Kumar et al., 2018; Martinez et al., 2008; Terron-Canedo et al., 2016). Researchers have previously investigated the effect of HPV 16 *E5* on host miRNA expression profiles and conducted integration analysis of mRNA and miRNA expression (Greco et al., 2011). They focused on *hsa-miR-146a, miR-203*, and *miR-324-5p* and their potential functions. However, whether BPV *E5*, the most significant transforming oncogene of BPV, influences host cell miRNA expression profiles and its role in regulating gene expression remains largely unknown.

Many algorithms have been developed for the prediction of miRNA-mRNA interactions. For example, the miRanda algorithm is based on a comparison of miRNAs complementarity to 3′ UTR regions. The binding energy of the duplex structure, evolutionary conservation of the whole target site and its position within 3′ UTR are used to assess the predicted targets (John et al., 2004; Witkos, Koscianska & Krzyzosiak, 2011). The TargetScan algorithm requires perfect complementarity to the seed region of a miRNA and then extends these regions to unravel complementarity outside the region (Lewis et al., 2003). RNAhybrid is another tool for the easy, fast, and flexible prediction of miRNA targets (Kruger & Rehmsmeier, 2006). However, predictions are blind to cellular transcriptome and miRNA repertoire, and the binding patterns do not always adhere to canonical rules of base pairing. Experimental studies of miRNA interactions were enhanced by high-throughput sequencing of RNAs isolated by crosslinking immunoprecipitation (HITS-CLIP) (Chi et al., 2009). Later, a modified HITS-CLIP termed covalent ligation of endogenous Argonaute-bound RNAs (CLEAR)-CLIP was developed (Moore et al., 2015). Recently, CLEAR-CLIP was used to define the miRNA-target interaction landscape in bovine kidney cells, providing a significant resource for understanding bovine and species-conserved miRNA regulation (Scheel et al., 2017).

In the present study, we constructed stable Madin-Darby bovine kidney (MDBK)-GFP-E5 cells and MDBK-GFP cells expressing the BPV-13 *E5* gene and *GFP* gene, respectively. Then we performed small RNA sequencing and transcriptome sequencing to explore alterations in miRNAs and their target genes in BPV E5 overexpressing MDBK cells compared with the negative controls to provide new insights into the mechanisms of BPV *E5* transformation of host cells.

## Materials and Methods

### Cell culture

MDBK (NBL-1) cells were purchased from the Cell Bank of the Chinese Academy of Sciences (Shanghai, China). They were cultured in RPMI-1640 medium with 10% fetal bovine serum (Grand Island, NY, USA) supplemented with 100 U/mL penicillin and 100 mg/mL streptomycin (Grand Island, NY, USA) at 37 °C in a 5% CO_2_ incubator.

### Lentivirus packaging

The BPV-13 *E5* fragment (GenBank accession no. KM258443.2, Pang et al., 2014) was cloned into the *Asis*I and *Mlu*I sites of lentiviral plasmid pLent-EF1a-FH-CMV-GFP-P2A-Puro (Vigenebio, Shandong, China). The C-terminus of *E5* was fused with a flag-6×his tag (Fig. S1). This recombinant plasmid pLent-EF1a-E5-FH-CMV-GFP-P2A-Puro and the control plasmid were co-transfected with PMD2G and PSPAX2 packaging plasmids into HEK293T cells, respectively. After 72 h, the supernatant was collected for purification by ultracentrifugation. The purified recombinant lentivirus and control lentivirus with a high-titer of 1.0 × 10^8^ TU/mL (transducing units/milliliter) were collected and stored at −70 °C.

### Establishment of a stable MDBK cell line expressing *E5*

MDBK cells in a 24-well plate were infected with recombinant lentivirus and negative lentivirus at a multiplicity of infection of 50. After 3 days, cells were subcultured and selected by puromycin (Solarbio, Beijing, China) at two μg/mL. Approximately 12 days later, polyclonal stable MDBK cell lines expressing *E5* and *GFP* (MDBK-GFP-E5) or *GFP* alone (MDBK-GFP) were obtained. BD FACSCalibur (BD Biosciences, San Jose, CA, USA) was used to detect the percentage of positive cells stably expressing *GFP* gene in MDBK-GFP and MDBK-GFP-E5 cells. Immunoprecipitation was used to detect the fused E5 protein using monoclonal anti-FLAG M2 antibody (Sigma-Aldrich, Shanghai, China). Briefly, around two μg monoclonal anti-FLAG M2 antibody (Sigma-Aldrich, Shanghai, China) was added to one mg cell lysate of MDBK-GFP as well as MDBK-GFP-E5 and gently rotated at 4 °C overnight. Next, 50 μl protein A+G agarose beads (Beyotime, Shanghai, China) was added to each sample and rotated at 4 °C for 2 h. Immunoprecipitated complexes were collected by centrifugation and pellets were washed five times with 0.5 mL RIPA buffer. The pellets were resuspended in 1× SDS-PAGE loading buffer and boiled for 10 min to dissociate the immunocomplexes from the beads. Finally, the supernatant was collected for Tricine-SDS-Page (Solarbio, Beijing, China) and western blotting. A mouse monoclonal anti-FLAG M2 primary antibody 1:2,000 and a goat anti-mouse IgG-HRP secondary antibody 1:5,000 (Santa Cruz Biotechnology, Santa Cruz, CA, USA) were used to detect E5-flag.

### RNA extraction

Total RNA was extracted from a polyclonal MDBK-GFP and MDBK-GFP-E5 cell line using an Ambion mirVana miRNA Isolation Kit (Thermo Fisher Scientific, Waltham, MA, USA). The quality of total RNA was analyzed using Bioanalyzer 2100 (Agilent Technologies, Santa Clara, CA, USA), and the concentration of the total RNA was quantified with a NanoDrop 2000 (Thermo Fisher Scientific, Lafayette, CO, USA).

### RNA sequencing and small RNA sequencing

For each sample, two μg total RNA was used for cDNA library construction and paired-end sequencing using an Illumina HiSeq4000 platform by Gene Denovo Biotechnology Co. (Guangzhou, China). The same samples were used for single-end 50 bp (SE50) small RNA sequencing using an Illumina HiSeq 2500 platform by Gene Denovo Biotechnology Co. (Guangzhou, China). The differentially expressed (DE) mRNAs were selected with |log2(fold change)| ≥ 1 and FDR ≤ 0.05 as we described previously (Peng et al., 2018). The expression levels of miRNAs were normalized based on the read counts to tags per million counts and |log2(fold change)| ≥ 1 and *p* ≤ 0.05 were the cutoffs to determine DE miRNAs. The raw and processed data have been deposited into the Gene Expression Omnibus database (https://www.ncbi.nlm.nih.gov/geo/) under accession number GSE133614.

### Gene ontology analysis

Gene ontology has three ontologies: biological process, cellular component, and molecular function. The basic unit of GO is GO term. GO enrichment analysis presents the GO terms significantly enriched of differentially expressed genes (DEGs) compared with the reference genome. It is calculated by the hypergeometric test and *p* ≤ 0.05 is regarded as significant enrichment (Ashburner et al., 2000; Pang et al., 2019b). The formula is as below:

$$P = 1 - \sum\limits_{i = 0}^{m - 1} {{{\left( {\matrix{
   M  \cr 
   i  \cr 

 } } \right)\left( {\matrix{
   {N - M}  \cr 
   {m - i}  \cr 

 } } \right)} \over {\left( {\matrix{
   N  \cr 
   n  \cr 

 } } \right)}}} $$

*M*, The number of all genes annotated to certain GO terms; *m*, The number of DEGs in *M*; *N*, The number of all genes with GO annotation; *n*, The number of DEGs in *N*.

### Target genes prediction and miRNA-mRNA network construction

RNAhybrid (v2.1.2) (Kruger & Rehmsmeier, 2006), Miranda (v3.3a) (John et al., 2004), and TargetScan (Version: 7.0) (Lewis et al., 2003) were used at their default parameters to predict the target genes of DE miRNAs. The reversely correlated DE miRNAs and DEGs from RNA-seq were used to construct miRNA-mRNA networks using Cytoscape 3.6.0 software. Cytoscape is a free software package for visualizing and analyzing molecular and genetic interaction networks. In Cytoscape, nodes representing biological entities, such as proteins or genes, are connected with edges representing pairwise interactions, such as protein–protein interactions. A key feature of Cytoscape is its ability to set visual aspects of nodes and edges, such as shape, color, and size, based on attribute values (Cline et al., 2007).

### RT-qPCR validation of differentially expressed miRNAs and genes

For miRNA validation, total RNA from each sample was reverse transcribed using a TaqMan MicroRNA Reverse Transcription Kit (Applied Biosystems, Foster City, CA, USA) according to the manufacturer’s protocol. Then the cDNA was used for qPCR with a TaqMan Small RNA Assay (20×) on an ABI 7500 Real-Time PCR System (Applied Biosystems, Foster City, CA, USA). *U6* snRNA was used as an internal control for normalization of the expression levels of miRNAs. RT-qPCR validation of DEGs was conducted as previously described (Pang et al., 2019a). All experiments were performed independently three times.

### Statistical analysis

Statistical significance analysis was performed by Student’s *t*-test, and *p* ≤ 0.05 was considered statistically significant. Data are presented as means ± SD from three independent experiments.

## Results

### Establishment of a stable MDBK cell line expressing bovine papillomavirus *E5* gene

**Figure 1:**
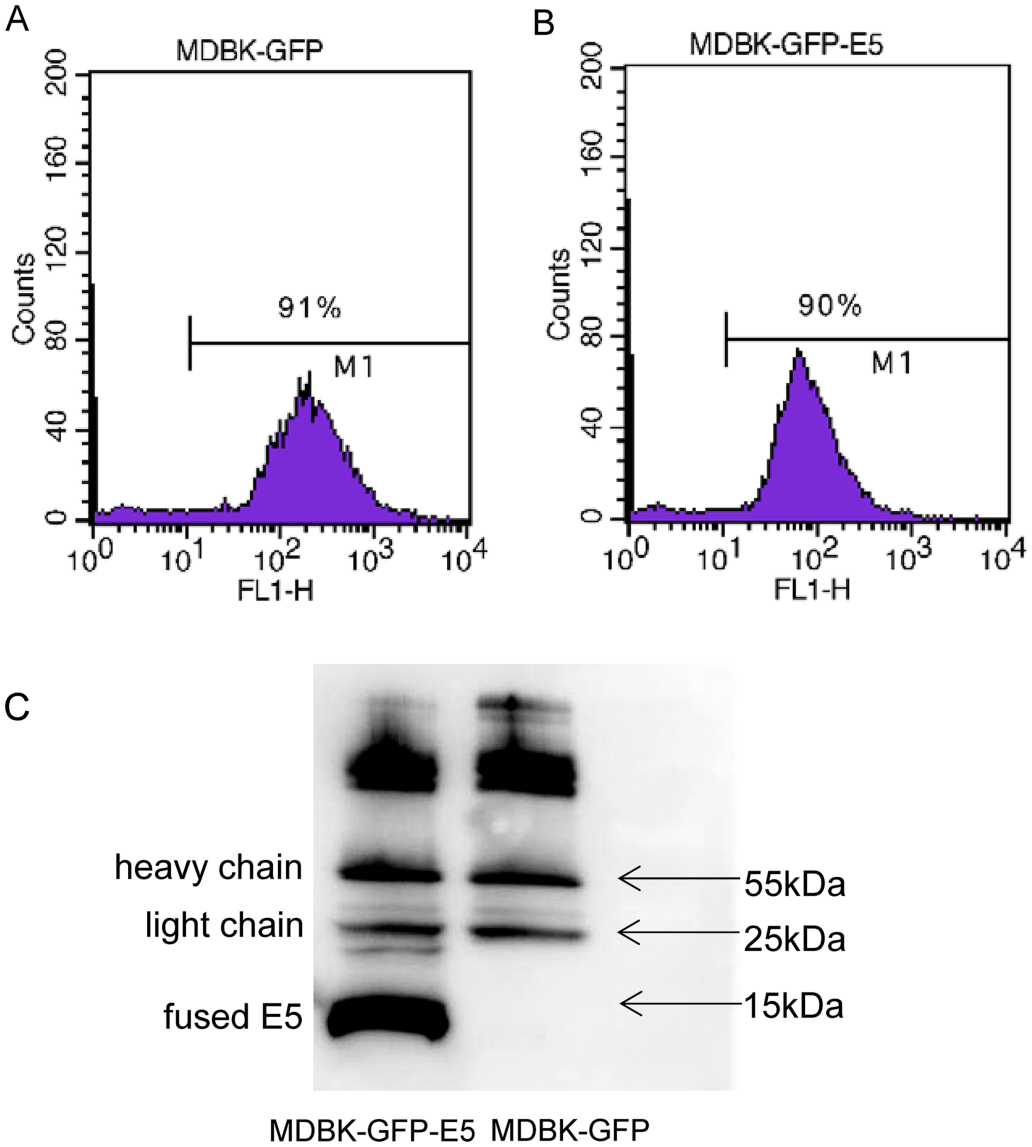
Generation of MDBK-GFP and MDBK-GFP-E5 stable cell lines. (A and B) Flow cytometric analysis of GFP-positive MDBK cells stably expressing GFP alone or GFP-E5. *Y*-axis represents cell counts; *X*-axis represents green fluorescence intensity; 10^0^–10^1^ represents negative cells, >10^1^ represents positive cells expressing *GFP*. (C) IP analysis of BPV E5 gene expression. The 55 and 25 kDa bands represent the heavy and light chain of the mouse monoclonal Flag antibody, respectively. The bottom band of less than 15 kDa in size represents the E5-flag protein.

Flow cytometric analysis indicated that there were 91% and 90% positive cells expressing GFP in MDBK-GFP and MDBK-GFP-E5 stable cell lines, respectively (Figs. 1A, 1B). The E5 protein is a strong transmembrane protein, therefore, immunoprecipitation was used to enrich and detect the E5 protein fused with Flag and his tags. The fused E5 protein could be detected at around 15 kDa in MDBK-GFP-E5 sample but it could not be detected in the MDBK-GFP sample. However, the light chain (approximately 25 kDa) and heavy chain (approximately 55 kDa) were detected in both MDBK-GFP cells and MDBK-GFP-E5 cells (Fig. 1C; Fig. S2).

### Analysis of DEGs and GO enrichment analysis

**Figure 2:**
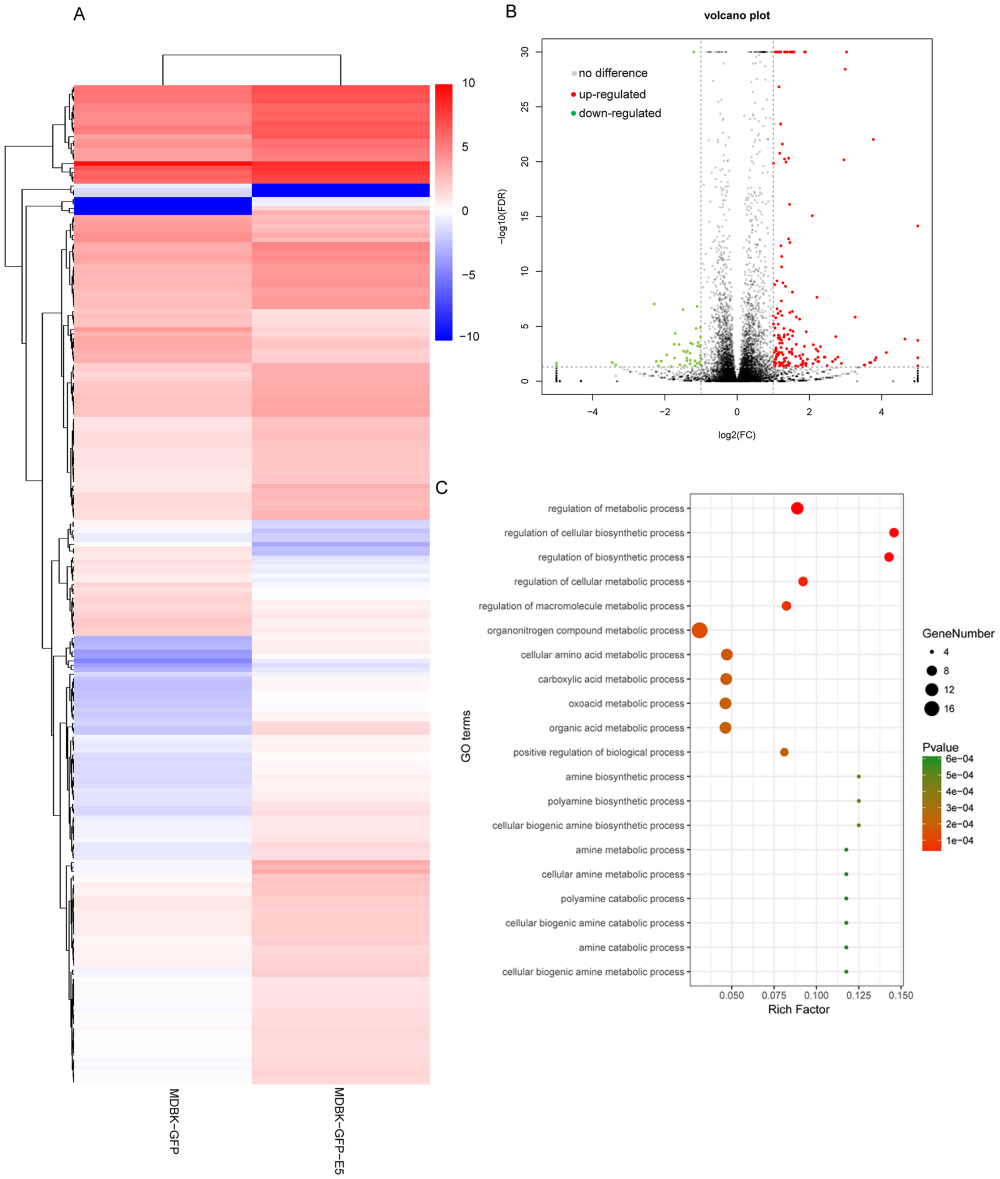
Analysis of differentially expressed genes in MDBK-GFP-E5 cells compared with MDBK-GFP cells. (A) Heatmap of the upregulated and downregulated genes. The color represents log_2_(FPKM) of genes. (B) Volcano plot of DEGs. Vertical lines correspond to twofold changes in upregulation and downregulation. Horizontal line represents *FDR* 0.05. The red and green dots represent upregulated and downregulated genes, respectively, while the gray dots represent genes with no significant difference. (C) The top 20 significantly enriched GO terms in “Biological Process”. *Y*-axis represents GO terms; *X*-axis represents rich factor; (rich factor equals the ratio between the differentially expressed genes and all annotated genes enriched in the GO term); the color and size of each bubble represent enrichment significance and the number of genes enriched in a GO term, respectively.

According to the criteria log2(fold change)| ≥ 1 and FDR ≤ 0.05, 223 DEGs were identified in the MDBK-GFP-E5 sample compared with the MDBK-GFP sample. Among these, 175 genes were up-regulated and 48 genes were down-regulated (Figs. 2A and 2B; Table S1). Obviously, the number of up-regulated genes was much more than that of the down-regulated genes. Next, we performed GO enrichment analysis of these DEGs. The top 20 significantly enriched GO terms in biological process ontology were predominantly involved in metabolic and biosynthetic processes, including the organonitrogen compound metabolic process, cellular amino acid metabolic process, organic acid metabolic process, cellular biogenic amine biosynthetic process (Fig. 2C).

### Analysis of differential expression of miRNAs

**Figure 3:**
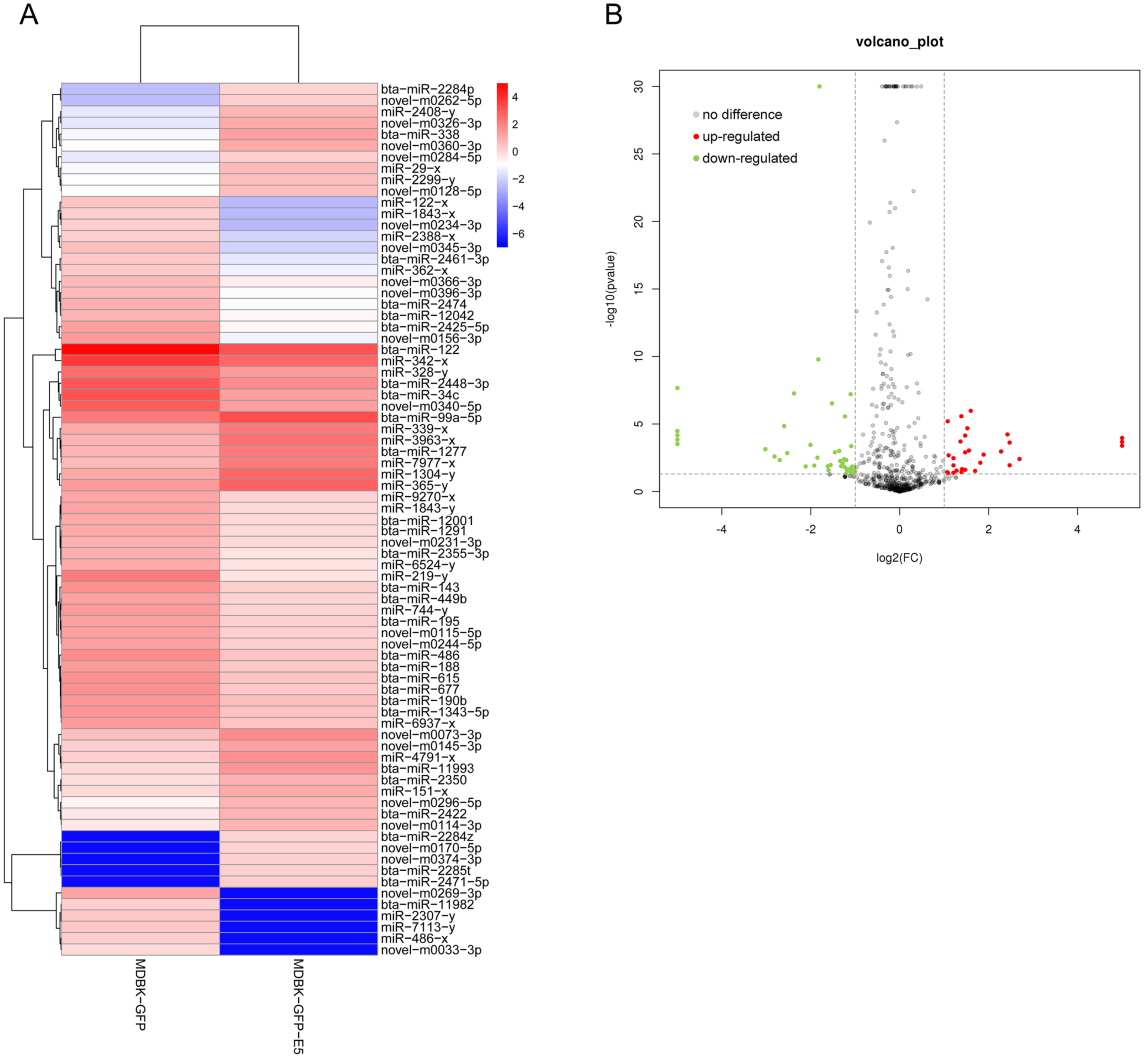
Analysis of differentially expressed miRNAs in MDBK-GFP-E5 cells compared with MDBK-GFP cells. (A) Heatmap of the upregulated and downregulated miRNAs. The color represents log_2_(FPKM) of miRNAs. (B) Volcano plot of dysregulated miRNAs. Vertical lines correspond to twofold changes in up-regulation and down-regulation. Horizontal line represents *p* ≤ 0.05. The red and green dots represent up-regulated and down-regulated miRNAs, respectively while the gray dots represent miRNAs with no significant difference.

Through small RNA sequencing, 31 miRNAs were identified as up-regulated and 46 miRNAs were identified as down-regulated according to the criteria |log2(fold change)| ≥ 1 and *p* ≤ 0.05 (Figs. 3A and 3B). Among these, 30 miRNAs (20 down-regulated, 10 up-regulated) were bovine-derived existing in miRBase 22.0 (Table 1). MiRNAs with -*x* or -*y* indicates isomiRs, the sequence-variants of miRNAs derived from the 5′ arm or 3′ arm of miRNA precursors, respectively. Furthermore, 22 miRNAs were predicted as novel miRNAs. Sequences of all known and novel miRNAs in the MDBK-GFP-E5 sample and the MDBK-GFP-E5 sample are listed in Table S2.

**Table 1:** A total of 30 bovine-derived differentially expressed miRNAs.

Upregulated miRNAs	*p*	log2(FC)	Downregulated miRNAs	*p*	log2(FC)
*bta-miR-1277*	0.000199	1.3658253	*bta-miR-122*	6.28E-33	−1.80465
*bta-miR-2284p*	0.011409	2.47097437	*bta-miR-1291*	0.033241	−1.11421
*bta-miR-2284z*	0.000404	6.68509917	*bta-miR-1343-5p*	0.014174	−1.06828
*bta-miR-2285t*	0.000109	6.90749159	*bta-miR-143*	0.001248	−1.45815
*bta-miR-2350*	0.043061	1.0874537	*bta-miR-188*	0.018018	−1.06524
*bta-miR-2422*	0.028417	1.27809491	*bta-miR-190b*	0.015811	−1.02672
*bta-miR-2471-5p*	0.000109	6.90749159	*bta-miR-195*	0.02037	−1.11417
*bta-miR-338*	5.93E-05	2.42203576	*bta-miR-2355-3p*	0.029067	−1.18453
*bta-miR-99a-5p*	6.3E-06	1.0785	*bta-miR-2425-5p*	0.000355	−2.00731
*bta-miR-11993*	0.000925	1.555697	*bta-miR-2448-3p*	2.74E-06	−1.23231
			*bta-miR-2461-3p*	0.011999	−1.92145
			*bta-miR-2474*	0.003129	−1.85111
			*bta-miR-34c*	1.65E-10	−1.83031
			*bta-miR-449b*	0.008176	−1.28403
			*bta-miR-486*	0.000985	−1.36204
			*bta-miR-615*	0.004733	−1.20159
			*bta-miR-677*	0.004071	−1.25169
			*bta-miR-12042*	0.010876	−1.55484
			*bta-miR-11982*	0.000069	−6.91468
			*bta-miR-12001*	0.022754	−1.17833

### miRNA target genes prediction and miRNA-gene network construction

**Figure 4:**
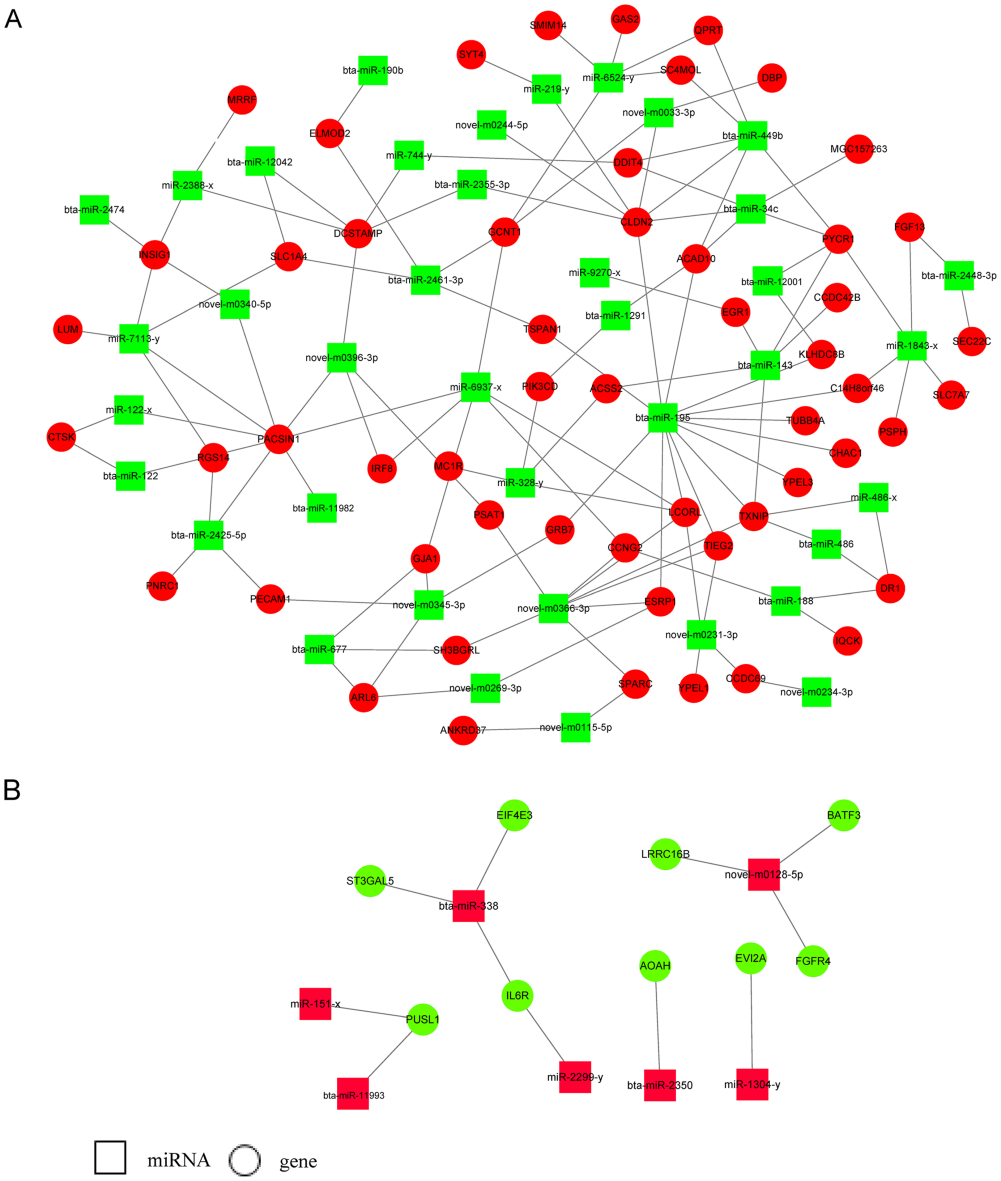
miRNA-gene regulatory networks based on upregulated miRNAs and downregulated genes or downregulated miRNAs and upregulated genes. (A) ****A miRNA-gene regulatory network based on downregulated miRNAs and upregulated genes. (B) A miRNA-gene regulatory network based on upregulated miRNAs and downregulated genes. Red and green represent up- and down-regulation, respectively. Rectangle and Round represent miRNAs and genes, respectively.

Three target prediction tools, RNAhybrid, Miranda, and TargetScan, were used to find potential miRNA and mRNA interactions. The intersection of the results was more reliable. Because miRNAs functioned as negative regulators repressing or degrading their targets, only miRNAs and their target genes with reverse expression namely upregulated miRNAs and downregulated target genes or downregulated miRNAs and upregulated target genes were used for constructing the miRNA-gene regulatory network in E5 over-expressing MDBK cells (Fig. 4; Table S3). We found that some miRNAs had multiple target genes. For example, *bta-miR-195* has more than 10 candidate targets; novel-m0366-3p potentially binds to eight target genes and miR-1843-x potentially binds to five targets. We also found that one target could interact with several miRNAs. For instance, *CLDN2* was predicted to interact with seven miRNAs; *INSIG1* potentially interacted with bta-miR-2474, miR-2388-x, miR-7113-y, and novel-m0340-5p; *SLC1A4* was predicted to bind to bta-miR-2461-3p, miR-7113-y and novel-m0223-5p. Furthermore, *bta-miR-449b* and *bta-miR-34c* shared four targets: *ACAD10*, *DDIT4*, *PYCR1*, and *CLDN2*. The results indicated that a potential regulatory network existed in BPV E5 over-expressing MDBK cells.

### RT-qPCR validation of DE miRNAs and DEGs s in a MDBK-GFP-E5 sample compared with a MDBK-GFP sample

**Figure 5:**
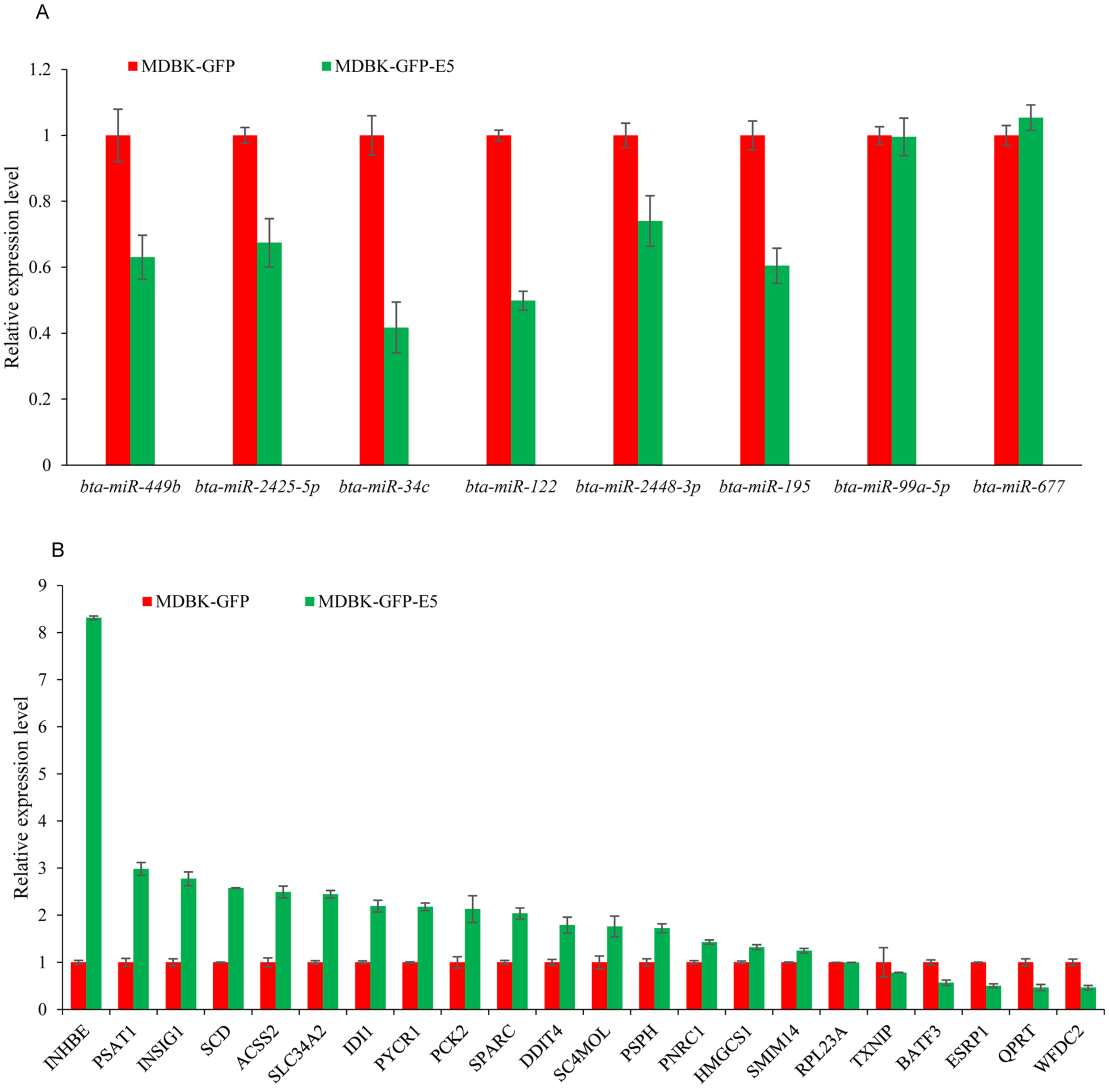
RT-qPCR validation of DE miRNAs and DEGs. (A) RT-qPCR validation of the selected miRNAs. (B) RT-qPCR validation of the selected DEGs. Data from RT-qPCR assays are the means of three independent replicates, with error bars representing SD.

To investigate the credibility of the high-throughput sequencing, nine miRNAs: *bta-miR-34c*, *bta-miR-99a-5p*, *bta-miR-122*, *bta-miR-143*, *bta-miR-195*, *bta-miR-449b*, *bta-miR-677*, *bta-miR-2425-5p*, and *bta-miR-2448-3p* were randomly selected for RT-qPCR validation. As a consequence, six miRNAs namely *bta-miR-34c*, *bta-miR-122*, *bta-miR-195*, *bta-miR-449b*, *bta-miR-2425-5p*, and *bta-miR-2428-3p* were downregulated, consistent with the small RNA-seq data, while *bta-miR-143* could not been amplified by Taqman probes (Fig. 5A). Furthermore, 22 DEGs namely *ACSS2* (acyl-CoA synthetase short-chain family member 2), *DDIT4* (DNA-dama ge-inducible transcript 4), INHBE (inhibin, beta E), *INSIG1* (insulin induced gene 1), *PNRC1* (proline-rich nuclear receptor coactivator 1), *PSAT1* (phosphoserine aminotransferase 1), *PSPH* (phosphoserine phosphatase), *PYCR1* (pyrroline-5-carboxylate reductase 1), *QPRT* (quinolinate phosphoribosyltransferase), *SC4MOL* (methylsterol monooxy-genase 1), *SLC34A2* (solute carrier family 34, member 2), *SCD* (stearoyl-CoA desaturase (delta-9-desaturase), *IDI1* (isopentenyl-diphosphate delta isomerase 1), *PCK2* (phosphoenolpyruvate carboxykinase 2), *SPARC* (Secreted protein acidic and cysteine rich), *HMGCS1* (3-hydroxy-3-methylglutaryl-CoA synthase 1), *SMIM14* (small integral membrane protein 14), *RPL23A* (60S ribosomal protein L23a), *TXNIP* (thioredoxin interacting protein), *BATF3* (basic leucine zipper transcription factor, ATF-like 3), *ESRP1* (epithelial splicing regulatory protein 1), *WFDC2* (WAP four-disulfide core domain 2), (FPKM > 10 in MDBK-GFP and/or MDBK-GFP-E5 group, Table S1) were randomly selected for RT-qPCR validation. The qPCR primers were listed in Table 2. Except for *QPRT*, *RPL23A*, *TXNIP*, *ESRP1*, the remaining 18 genes were all consistent with RNA-seq results (Fig. 5B).

**Table 2:** List of qPCR primers for DEGs validation.

Gene symbol	Primer sequence(5′-3′)	Gene symbol	Primer sequence(5′-3′)
*GAPDH*	F:TGAAGGTCGGAGTGAACGGA	*INHBE*	F:GCTGTCGGCGAGACCATTAT
	R:TGCCGTGGGTGGAATCATAC		R:GAAGTACCCAAGGGCCAAGG
*PYCR1*	F:CGACACGTACGGGAGCC	*SMIM14*	F:TCTGGGCGGATTTAAACAGTCA
	R:GGTTCACCCCCATCTTCCTG		R:TGCAATAACCATCCAGGCCA
*SC4MOL*	F:CGGTCTGGCGGCAGAATAATA	*PSAT1*	F:TCCAGGCCAGTGGATGTTTC
	R:ATGAGAGATCCCCACGTTGC		R:CAAACCCCAGCAGGTCATCT
*IDI1*	F:TTGCACCGAGCTTTCAGTGT	*SPARC*	F:ACGACAAGTACATCGCCCTG
	R:TTCCAGCTCACTTGGGTTGC		R:CTTGACCCCAGGCAGAACAA
*QPRT*	F:GGGAGGCCCTTCTTAGATGC	*SCD*	F:CTGCCATCGGTCTGGCTTAT
	R:GTAGTCTTCCTCGTGCCTGC		R:AAAGGAACCCAAGGGACCAC
*INHBE*	F: GCTGTCGGCGAGACCATTAT	*HMGCS1*	F:AAAGCTAGTGGCGGAGTGAA
	R: GAAGTACCCAAGGGCCAAGG		R:ATGGTGAAAGAACCAAATATGACCG
*DDIT4*	F:GCAAAGAACTACTGCGCCTG	*BATF3*	F:CACAGCAGTCTCAACAGAGCC
	R:GGCAGAGCTAAACAACCCCT		R:TCTGAGCAGCAACTCGGTTT
*INSIG1*	F:GTGACGGTGGGGAACATAGG	*RPL23A*	F:GCTGAAGCCAAAGCAAAGGC
	R: TCACTACCCAGTATCGCGGA		R:TCTTCCgAGGGTATTTGGGC
*PCK2*	F:CAACTCTCGCTTTTGTGCCC	*SLC34A2*	F:ATCACCTCCCTGCTGATCCT
	R: ACGGCCTCCAAAGATGATGG		R:CCGATCAGTGGGGTCATAGC
*PNRC1*	F:ACTGAAGTGAGCCAAAAGGAGA	*ESRP1*	F:GGCCTGGCCTACAATACTGG
	R:GGTGGTTCCCATCCAGTGAC		R:GGCCTGGTCACTTGTGTGTA
*WFDC2*	F:ATGCCTAATGACAAGCCGGG	*TXNIP*	F:TGATCCCCAAAGCTGCCATT
	R:GCAACCATTGACGCAGCATT		R:GATTTTCTGCACCCGAAGGC
*ACSS2*	F:CCGGATGTGCAGATCTCCTG		
	R:CATGTAGCCCCCAACTGTGT		

## Discussion

Following the development of high-throughput sequencing technology, researchers have focused on small RNA sequencing and RNA-seq for BPV research. Yuan et al. (2008) used gene expression profiles to identify candidate genes involved in the pathogenesis of equine skin tumors induced by BPV-1 infection. Several genes enriched in inflammation and immunity, apoptosis and RNA transcription/metabolism were dysregulated in their study. Researchers also conducted miRNA sequencing in BPV type 1-transformed equine cells (Terron-Canedo et al., 2016). They found 206 DE mature miRNAs in equine fibroblasts (EqPalFs) transformed with the BPV-1 genome compared with control EqPalFs. This showed that miRNAs were involved in equine sarcoids and cell transformation. However, whether *E5* oncogene expression could alter the miRNA and mRNA expression profiles of MDBK cells has not been reported.

In the present study, we conducted RNA-seq for BPV-13 *E5* overexpressing MDBK cells to explore the effect of the BPV *E5* on cellular mRNA expression profiles. We identified 223 DEGs in BPV *E5* overexpressing MDBK cells compared with control cells. The top 20 significantly enriched GO terms in “biological process” (*p* < 0.05) were associated with metabolic and biosynthetic processes. Among these DEGs, *PSPH*, and P*SAT1* which can be converted to serine and glycine, were both up-regulated. The serine and glycine pathways have important functions in proliferating cells and in inducing tumorigenesis (Antonov et al., 2014; Liu et al., 2016; Possemato et al., 2011). Furthermore, we found that *PYCR1* was also up-regulated in BPV *E5*-overexpressing cells. Previous studies have reported that *PYCR1*, a proline biosynthetic enzyme, was up-regulated in prostate cancer (PCa) compared with normal tissues and it could also promote PCa cell proliferation and colony formation (Nilsson et al., 2014; Zeng et al., 2017). The results revealed that BPV *E5* might induce cell transformation by altering the expression levels of genes involved in cellular metabolic and biosynthetic processes.

MicroRNAs are involved in a wide range of biological processes such as cell proliferation, apoptosis, immunity, and tumorigenesis (Cheng et al., 2005; Kumar et al., 2018; Martinez et al., 2008; Murakami et al., 2006). Moreover, several studies demonstrated that miRNAs play critical roles in virus-host interactions (Grassmann & Jeang, 2008; Scaria et al., 2006). In the present study, we focused on BPV *E5*, the most important oncogene of BPV and conducted small RNA sequencing for E5 overexpressing MDBK cells compared with control cells. Compared with the control, 77 miRNAs were DE. To identify the underlying miRNA regulatory mechanisms, an integrative analysis of the DE miRNAs and their predicted target genes was performed. We found that *PYCR1* was the common target of *bta-miR-34c*, *bta-miR-143*, and *bta-miR-449b*. It seemed that the three miRNAs might participate in cell transformation or tumorigenesis by post-transcriptional regulation of *PYCR1*. *DDIT4*, also known as *REDD1*, was a target shared by *bta-miR-34c* and *bta-miR-449b*. It is a cytoplasmic protein induced by DNA damage or other types of stress (Ellisen et al., 2002). Several in vivo and in vitro studies have demonstrated that *DDIT4* promotes cancer progression and angiogenesis (Dennis et al., 2013; Ellisen et al., 2002; Sofer et al., 2005). It was predicted that *bta-miR-34c* and *bta-miR-449b* could bind to and regulate the expression of *DDIT4*. We assumed that *bta-miR-34c* and *bta-miR-449b* might have critical roles in inducing cell transformation by BPV *E5*.

## Conclusions

In the present study, 77 miRNAs and 223 genes were DE following E5 over-expression. The DEGs were predominantly involved in metabolic and biosynthetic processes. A potential miRNA-gene regulatory network was constructed according to DE miRNAs and their predicted target genes. Six down-regulated miRNAs, *bta-miR-34c*, *bta-miR-122*, *bta-miR-195*, *bta-miR-449b*, *bta-miR-2425-5p*, and *bta-miR-2428-3p* were confirmed. A total of 16 upregulated genes: *ACSS2*, *DDIT4*, *INHBE*, *INSIG1*, *PNRC1*, *PSAT1*, *PSPH*, *PYCR1*, *SC4MOL*, *SLC34A2*, *SCD*, *SPARC*, *IDI1*, *PCK2*, *HMGCS1*, and *SMIM14* and two downregulated genes: *BATF3* and *WFDC2* were confirmed. Specially, *bta-miR-34c* and *bta-miR-449b* potentially regulated *PYCR1* and *DDIT4* which were involved in cancer progression and angiogenesis. Our study showed for the first time the comprehensive landscape of gene and miRNA alterations in BPV *E5*-overexpressing MDBK cells, providing new insights into the tumorigenesis mechanisms of the BPV *E5* oncogene.

## Supplemental Information

10.7717/peerj.8098/supp-1 Supplemental Information 1 The lentiviral plasmid map.

10.7717/peerj.8098/supp-2 Supplemental Information 2 IP analysis of BPV E5 gene expression. The 55 and 25 kDa bands represent the heavy and light chain of the mouse monoclonal Flag antibody, respectively. The bottom band of less than 15 kDa in size represents the E5-flag protein.

10.7717/peerj.8098/supp-3 Supplemental Information 3 Differentially expressed genes (DEGs) in MDBK-GFP-E5 group compared with MDBK-GFP group.

10.7717/peerj.8098/supp-4 Supplemental Information 4 Sequences of all known and novel miRNAs in MDBK-GFP-E5 sample and MDBK-GFP sample.

10.7717/peerj.8098/supp-5 Supplemental Information 5 Differentially expressed miRNAs and mRNAs with reverse expression used for constructing miRNA-mRNA regulatory network.

## Additional Information and Declarations

### Data Availability

The following information was supplied regarding data availability: The raw and processed data are available at GEO: GSE133614.
