# Supplementary figures and images for: Comprehensive analysis of differentially expressed microRNAs and mRNAs in MDBK cells expressing bovine papillomavirus E5 oncogene
Source: PeerJ. 2019 Nov 22;7:e8098. doi: 10.7717/peerj.8098 (PMC6876490; doi:10.7717/peerj.8098)

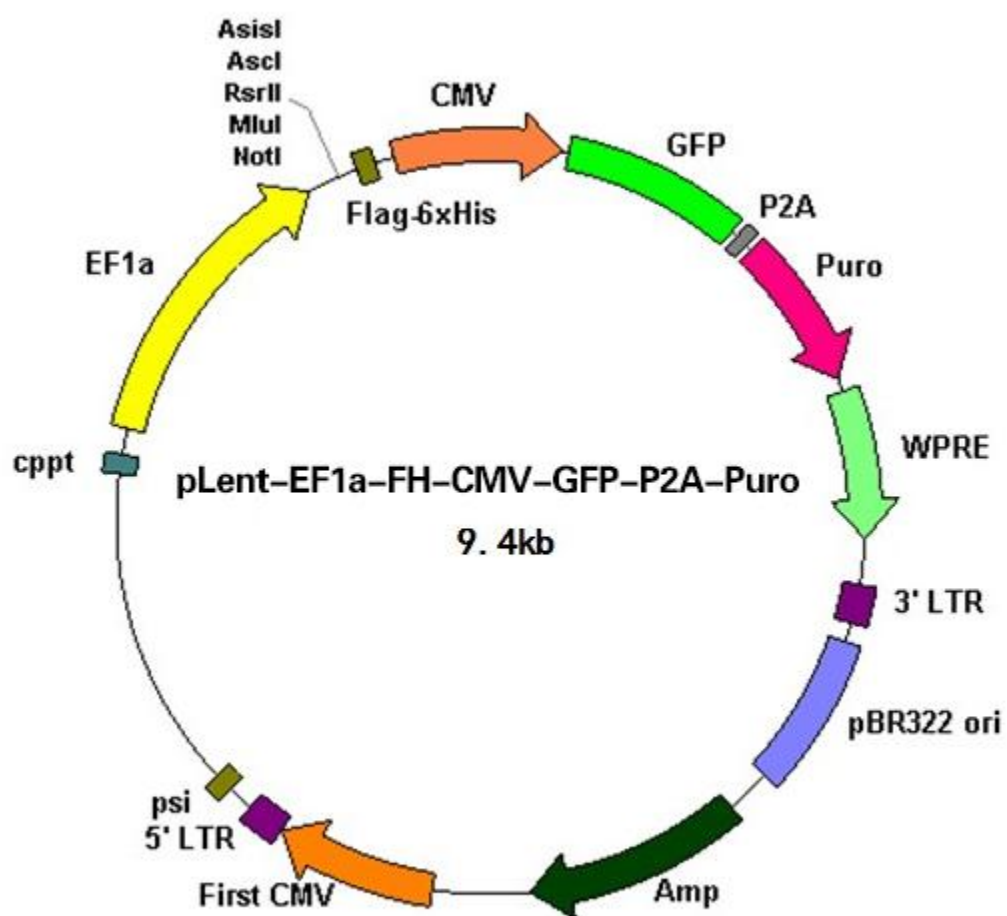

Supplement: Supplemental Information 1 [file peerj-07-8098-s001.pdf]

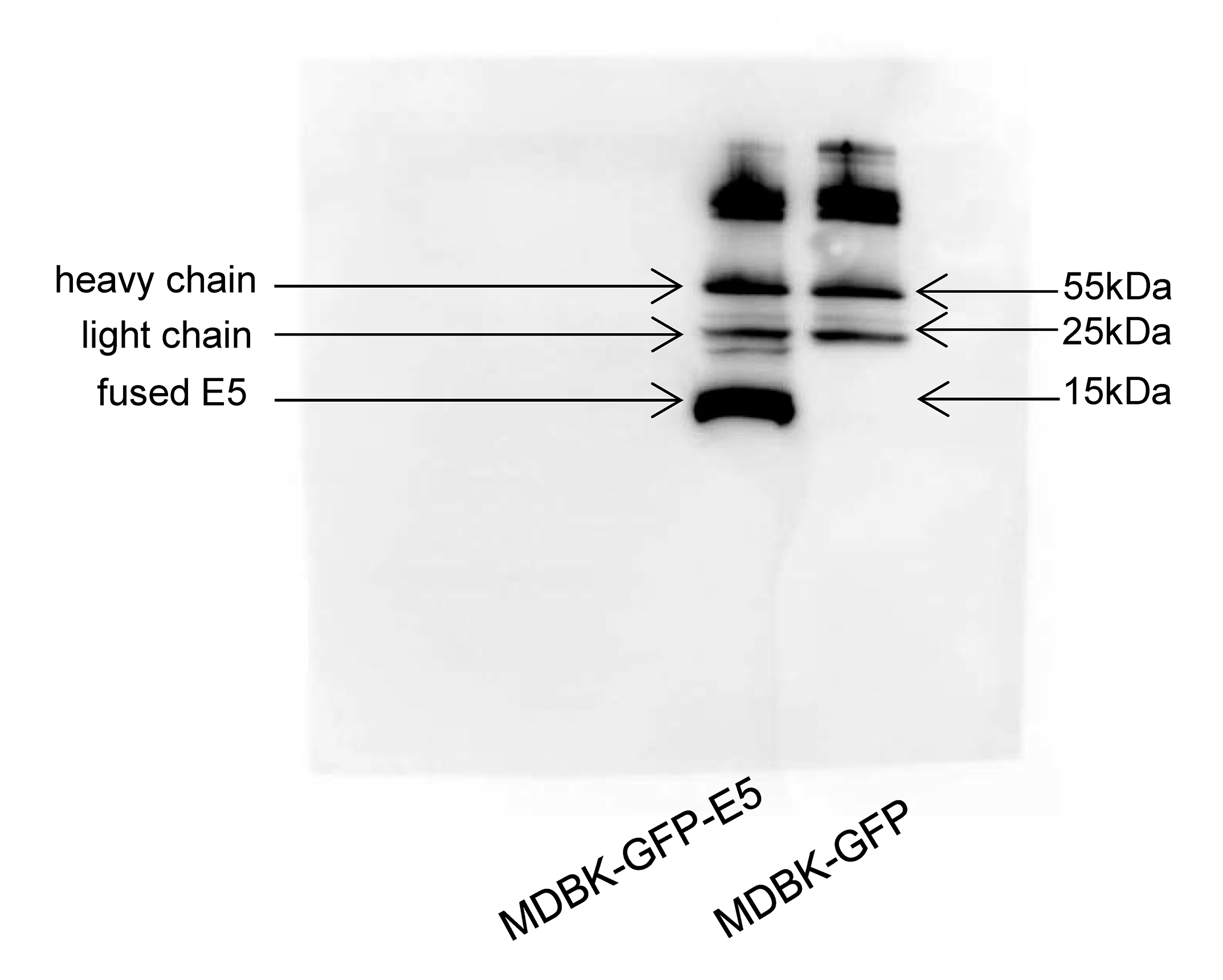

Supplement: Supplemental Information 2 [file peerj-07-8098-s002.png]
